# Supplementary material for: Characterization of CD4-Positive Lymphocytes in the Antiviral Response of Olive Flounder (Paralichthys oliveceus) to Nervous Necrosis Virus
Source: Int J Mol Sci. 2020 Jun 11;21(11):4180. doi: 10.3390/ijms21114180 (PMC7312829; doi:10.3390/ijms21114180)
Supplement: Supplementary file 1 [file ijms-21-04180-s001.pdf]

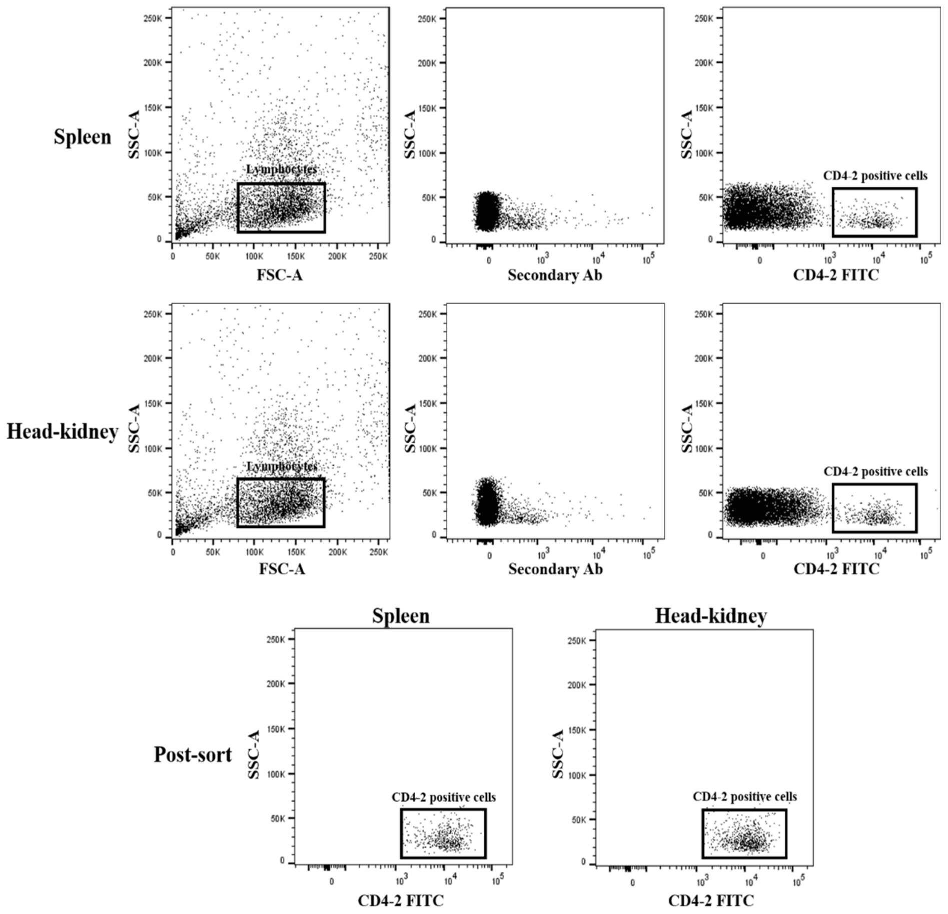

Supplemental Figure 1. Pre- and post-sort analysis of CD4-2 negative and CD4-2 positive lymphocytes. Leukocytes were obtained from spleen and head-kidney in olive flounder and lymphocytes were gated on FSC and SSC area. Cells treated only with secondary Ab (FITC) were represented in middle panel, and cells stained with anti-CD4-2 mAb (3C8) were shown in right panel.

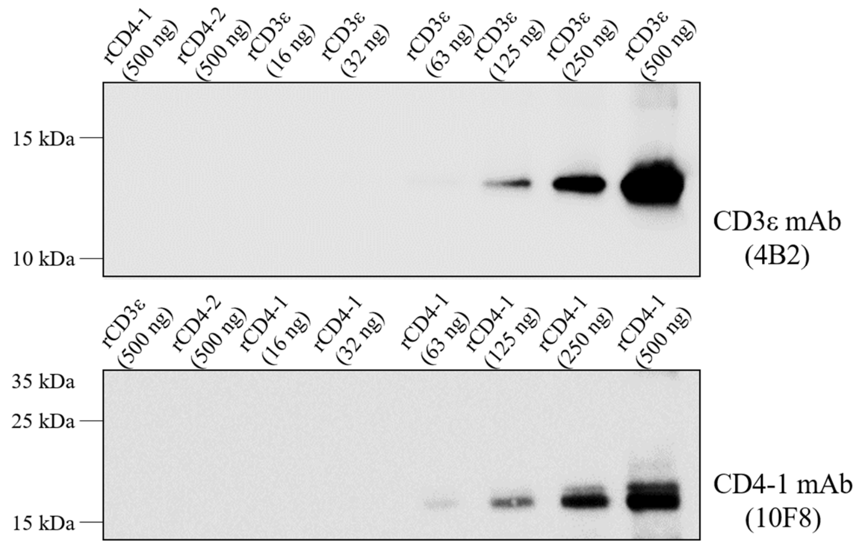

Supplemental Figure 2. Specificity of anti-CD3ε and CD4-1 mAbs. Increasing amounts of recombinant CD3ε and CD4-1 proteins were detected with anti-CD3ε and anti-CD4-1 mAbs in immunoblot analysis. The recombinant CD3ε protein reacted with the anti-CD3ε mAb (4B2) to produce a band of approximately ~ 15kDa, and the recombinant CD4-1 protein reacted with the anti-CD4-1 mAb (10F8-3) to produce a band with a molecular weight of approximately ~ 17 kDa.
